# Lansoprazole Alone or in Combination With Gefitinib Shows Antitumor Activity Against Non-small Cell Lung Cancer A549 Cells *in vitro* and *in vivo*

**DOI:** 10.3389/fcell.2021.655559

**Published:** 2021-04-20

**Authors:** Xiaoxia Zhao, Ning Zhang, Yingying Huang, Xiaojing Dou, Xiaolin Peng, Wei Wang, Zhe Zhang, Ran Wang, Yuling Qiu, Meihua Jin, Dexin Kong

**Affiliations:** ^1^Tianjin Key Laboratory on Technologies Enabling Development of Clinical Therapeutics and Diagnostics, School of Pharmacy, Tianjin Medical University, Tianjin, China; ^2^Department of Otorhinolaryngology Head and Neck, Institute of Otorhinolaryngology, Tianjin First Central Hospital, Tianjin, China; ^3^School of Medicine, Tianjin Tianshi College, Tianyuan University, Tianjin, China

**Keywords:** lansoprazole, gefitinib, lung cancer, combination, autophagy

## Abstract

Lansoprazole (Lpz) is an FDA-approved proton pump inhibitor (PPI) drug for the therapy of acid-related diseases. Aiming to explore the new application of old drugs, we recently investigated the antitumor effect of Lpz. We demonstrated that the PPI Lpz played a tumor suppressive role in non-small cell lung cancer (NSCLC) A549 cells. Mechanistically, Lpz induced apoptosis and G0/G1 cell cycle arrest by inhibiting the activation of signal transducer and activator of transcription (Stat) 3 and the phosphoinositide 3-kinase (PI3K)/Akt and Raf/ERK pathways. In addition, Lpz inhibited autophagy by blocking the fusion of autophagosomes with lysosomes. Furthermore, Lpz in combination with gefitinib (Gef) showed a synergistic antitumor effect on A549 cells, with enhanced G0/G1 cell cycle arrest and apoptosis. The combination inhibited Stat3 phosphorylation, PI3K/Akt and Raf/ERK signaling, affecting cell cycle-related proteins such as p-Rb, cyclin D1 and p27, as well as apoptotic proteins such as Bax, Bcl-2, caspase-3, and poly (ADP-ribose) polymerase (PARP). *In vivo*, coadministration with Lpz and Gef significantly attenuated the growth of A549 nude mouse xenograft models. These findings suggest that Lpz might be applied in combination with Gef for NSCLC therapy, but further evidence is required.

## Introduction

The tumor microenvironment plays a pivotal role in tumor malignancy. The acidic microenvironment strongly contributes to tumor progression by stimulating invasion and metastasis, inhibiting the immune surveillance of cancers, and conferring chemoresistance (Taylor et al., 2015; Ibrahim-Hashim and Estrella, 2019). As an ATP-dependent proton pump, the vacuolar-H^+^ ATPase (V-ATPase) seems to be involved in the acidification of the tumor microenvironment (Spugnini et al., 2015). The V-ATPase proton pump is a multisubunit membrane protein complex that is present in intracellular membranes, such as lysosomes, endosomes, and secretory vesicles, and in the plasma membranes of specialized cells (Stransky et al., 2016).

Proton pump inhibitors (PPIs) are prodrugs that are activated by acid and are currently used as anti-acid drugs for the treatment of acid-related diseases (Shi and Klotz, 2008; Shin and Kim, 2013); these disorders include peptic ulcer disease, gastroesophageal reflux disease, and idiopathic hypersecretion (Der, 2003). PPIs include omeprazole, esomeprazole, lansoprazole (Lpz), pantoprazole, and rabeprazole (Shi and Klotz, 2008). They act as potent inhibitors of gastric acid pumps and have been used for short- or long-term treatments with very high doses without major side effects (Der, 2003; Martín de Argila, 2005). Several studies have shown that PPIs have antitumor effects against different tumor types; for example, esomeprazole inhibits the proliferation of melanoma cells *in vitro* and reduces tumor growth in human melanoma engrafted mice (De Milito et al., 2010), and Lpz induces cell apoptosis in human breast cancer cells and attenuates tumorigenesis in MDA-MB-231 breast cancer cell xenografted mice (Zhang et al., 2014). In addition, Lpz showed most potent anti-tumor effect when compared to the other PPIs (omeprazole, esomeprazole, Lpz, pantoprazole, and rabeprazole) in human melanoma cells (Lugini et al., 2016); Among several PPIs (omeprazole, esomeprazole, Lpz, and pantoprazole), Lpz was the most effective to induce cell death in breast cancer cells (Zhang et al., 2014). However, the antitumor effects of Lpz in non-small cell lung cancer (NSCLC) have not yet been reported. Therefore, in the present study, we focused on the development of Lpz targeting acidic microenvironments in lung cancer.

Lung cancer is the most common cancer and the leading cause of cancer death in men and is the third most common cancer and the second leading cause of cancer death worldwide (Mao et al., 2016). Lung cancers are divided into two main groups: small-cell lung cancer (13% of the cases) and NSCLC (83% of the cases). Surgery is the major treatment modality for early stage NSCLC; cytotoxic chemotherapy remains a substantial part of therapy for most patients in locally advanced and metastatic stages, and targeted therapies have become standard therapies for patients with NSCLC (Rodriguez-Canales et al., 2016). Epidermal growth factor receptor (EGFR) is overexpressed in 40–80% of NSCLC cases (Rodriguez-Canales et al., 2016). Gefitinib (Gef, Iressa), a small molecule EGFR tyrosine kinase inhibitor, is one of the classical first-line treatment drugs that can benefit certain patients with EGFR-mutatant NSCLC. Consistently, Gef inhibits cell growth in PC-9 NSCLC cells with EGFR mutation, with a much higher potency than that in A549 and H226 NSCLC cells with wild type EGFR (Sugita et al., 2015). However, Gef also has adverse effects, such as hypersensitivity myocarditis related to Gef being the probable cause of death (Truell et al., 2005); adding pemetrexed and carboplatin chemotherapy to Gef significantly prolonged both progression-free and overall survival but also increased toxicity (Noronha et al., 2020). The drug combination could reduce the drug use concentration. Based on these theories, we extensively investigated the anticancer activity of Lpz and the antitumor synergistic effect of Lpz in combination with Gef in NSCLC A549 cells.

## Materials and Methods

### Cell Culture

A549 cells were obtained from the Cell Resource Center, Peking Union Medical College (Beijing, China). A549 cells have been authenticated using STR profiling within the last 3 years and A549 cells were confirmed by PCR to be free of mycoplasma contamination. A549 cells were cultured in RPMI 1640 medium containing 10% fetal bovine serum (FBS), 100 U/ml penicillin, and 100 μg/ml streptomycin. Cell cultures were maintained in a humidified atmosphere with 5% CO_2_ at 37°C.

### Reagents

Lansoprazole and gefitinib were purchased from Selleck Chemicals (Houston, TX, United States) and Target Molecule Corp. (Boston, MA, United States), respectively. Monodansylcadaverine (MDC) and propidium iodide (PI) were obtained from Sigma-Aldrich (St. Louis, MO, United States). A FITC-Annexin V apoptosis detection kit was obtained from BD Bioscience (San Josè, CA, United States). RPMI 1640 and FBS were purchased from the Biological Industries (Beit Haemek, Israel). Enhanced chemiluminescence (ECL) reagent was purchased from Thermo Fisher Scientific (Waltham, MA, United States). Antibodies specific for p27, p-Rb, cyclin D1, LC3B, p-signal transducer and activator of transcription (Stat) 3, phosphatidylinositol 3-kinase (PI3K)-p110α, PI3K-p110β, p-mammalian target of rapamycin complex 1 (mTORC1) (Ser2448), Akt, p-Akt (Ser473), p-p70 S6 kinase (S6K) (Thr389), p-glycogen synthase kinase 3 (GSK-3)β, poly (ADP-ribose) polymerase (PARP), caspase-3, K-Ras, p-extracellular signal-regulated kinase (ERK)1/2, p-c-Raf, Ki67, β-actin, and horseradish peroxidase-conjugated goat anti-rabbit and horse anti-mouse secondary antibodies were purchased from Cell Signaling Technology, Inc. (Danvers, MA, United States). Antibodies specific for Bcl-2 and Bax were obtained from Santa Cruz Biotechnology, Inc. (Dallas, TX, United States).

### Determination of Cell Viability

Cell viability was assessed using the MTT assay as we previously reported, with a small modification (Zhou et al., 2016). Briefly, A549 cells were plated in 96-well culture plates and incubated with various concentrations of Lpz for 48 h, and then 20 μl of MTT (5 mg/ml) was added to each well. After 4 h of incubation, the formazan was dissolved in DMSO, and the optical density (OD) at 490 nm was measured using an iMark microplate reader (Bio-Rad, Hercules, CA, United States).

### Flow Cytometric Analysis

The effects of Lpz and Gef on cell cycle distribution and apoptosis in A549 cells were analyzed by flow cytometry. Briefly, A549 cells were seeded in six-well plates and treated with Lpz for 48 h. For cycle analysis, cells were collected and fixed with 75% ethanol at 4°C overnight and stained with PI solution (25 μg/ml). The treated cells were analyzed with a BD Accuri C6 flow cytometer (BD Biosciences, San Jose, CA, United States).

For cell apoptosis analysis, Annexin V-FITC/PI double staining was used as reported by us previously with small modifications (Zhang et al., 2019). After harvesting, the cells were resuspended in binding buffer and incubated with Annexin V-FITC/PI solution in the dark for 15 min. Finally, samples were analyzed using a BD Accuri C6 flow cytometer.

Data were quantified with Flow Jo Software (Tristar, Long Beach, CA, United States).

### Measurement of Intracellular Reactive Oxygen Species (ROS) Levels

Intracellular reactive oxygen species (ROS) levels were determined as we reported previously with a small modification (Zhang et al., 2019). The ROS assay kit (Beyotime Biotechnology, China) was used. Briefly, A549 cells were plated in six-well culture plates and treated with various concentrations of Lpz for 24 h. Following the treatment, the cells were harvested and then incubated with 10 μM of 2′,7′-dichlorofluorescein diacetate (DCFH-DA) for 30 min at 37°C. The resulting fluorescent intensity was measured using a BD Accuri C6 flow cytometer.

### Wound Healing Assay

The wound healing assay was performed as we reported previously with a small modification (Wang et al., 2018). A549 cells were plated in 24-well plates and incubated overnight to a density of 70–80% in RPMI 1640 medium. Cell monolayers were mechanically wounded with a pipette tip and washed with PBS to remove debris. Then, different concentrations of Lpz were added to cells cultivated with fresh culture containing 2% FBS for 48 h. The wound areas were imaged with a microscope.

### Protein Extraction and Western Blotting

Western blot analysis was carried out as we previously reported with small modifications (Shao et al., 2018). Cells were collected with lysis buffer, and the protein concentration of each sample was determined using a BCA protein assay kit. Equal amounts of proteins were separated by sodium dodecyl sulfate-polyacrylamide gel electrophoresis (SDS-PAGE) and were subsequently transferred to PVDF membranes. After being blocked with 5% non-fat milk, the membranes were exposed to specified primary antibodies overnight at 4°C and then incubated with the respective HRP-conjugated secondary antibody for 1 h at room temperature. The binding was visualized by ECL reagents on a Bio-Rad ChemiDoc^TM^ XRS + System (Bio-Rad, Hercules, CA, United States).

### Monodansylcadaverine (MDC) Staining

Monodansylcadaverine, a specific marker for autophagic vacuoles, was used to measure whether Lpz induces autophagy. A549 cells were seeded in six-well plates on coverslips overnight, and Lpz was administered for 48 h. The cells were washed with ice-cold PBS and incubated with MDC (50 μM) for 30 min in the dark at 37°C. Then, the cells were washed twice with PBS, fixed in 4% paraformaldehyde for 10 min, and washed again with PBS. The slides were observed by fluorescence microscopy (BX51, Olympus, Japan).

### Analysis of Autophagic Flux

To analyze autophagic influx, A549 cells were transfected with a tandem fluorescent mRFP-GFP-LC3 plasmid using Lipofectamine 2000 according to the manufacturer’s instructions. The transfected cells were treated with Lpz for 24 h. The expression of GFP and mRFP was visualized with an Olympus FV1000 laser scanning confocal microscope (Olympus, Tokyo, Japan). Images were acquired using FV10-ASW3.0 software. Autophagic flux was evaluated by the color change of GFP/mRFP.

### Nude Mouse Xenograft Tumor Experiments

To establish xenograft tumors *in vivo*, individual mice were injected subcutaneously with A549 cells. When subcutaneous tumors grew to 30 to 50 mm^3^, the mice were randomized into four groups and treated with Lpz (25 mg/kg) or Gef (80 mg/kg) alone or in combination (Lpz was administered orally 2 h before Gef) every other day for 19 days. The growth of implanted tumors was monitored every other day, and the tumor volumes were calculated. Their body weights were also measured every other day. Mice were sacrificed after 19 days of treatment, and the tumors were excised.

Tumors were fixed in paraformaldehyde for immunohistochemistry (IHC) analysis. For IHC analysis, 5-μm paraffin sections underwent dewaxing, and endogenous peroxidase was blocked. The sections were heated in an antigen retrieval solution and were then incubated with Ki67 antibody overnight at 4°C, and the bound antibodies were detected with Bond Polymer (anti-rabbit poly-HRP-IgG) and visualized using a diaminobenzidine (DAB) peroxidase substrate. The images were collected using O8 microscope and slide scanner (Precipoint, Germany).

### Statistical Analysis

All data are expressed as the means ± SD of triplicate values. One-way ANOVA followed by Tukey’s multiple comparison test was utilized to determine the statistical significance with GraphPad Prism 5 (GraphPad, San Diego, CA, United States). Differences were considered statistically significant when *p* < 0.05.

## Results

### Antitumor Activity of Lpz in A549 Cells

**FIGURE 1:**
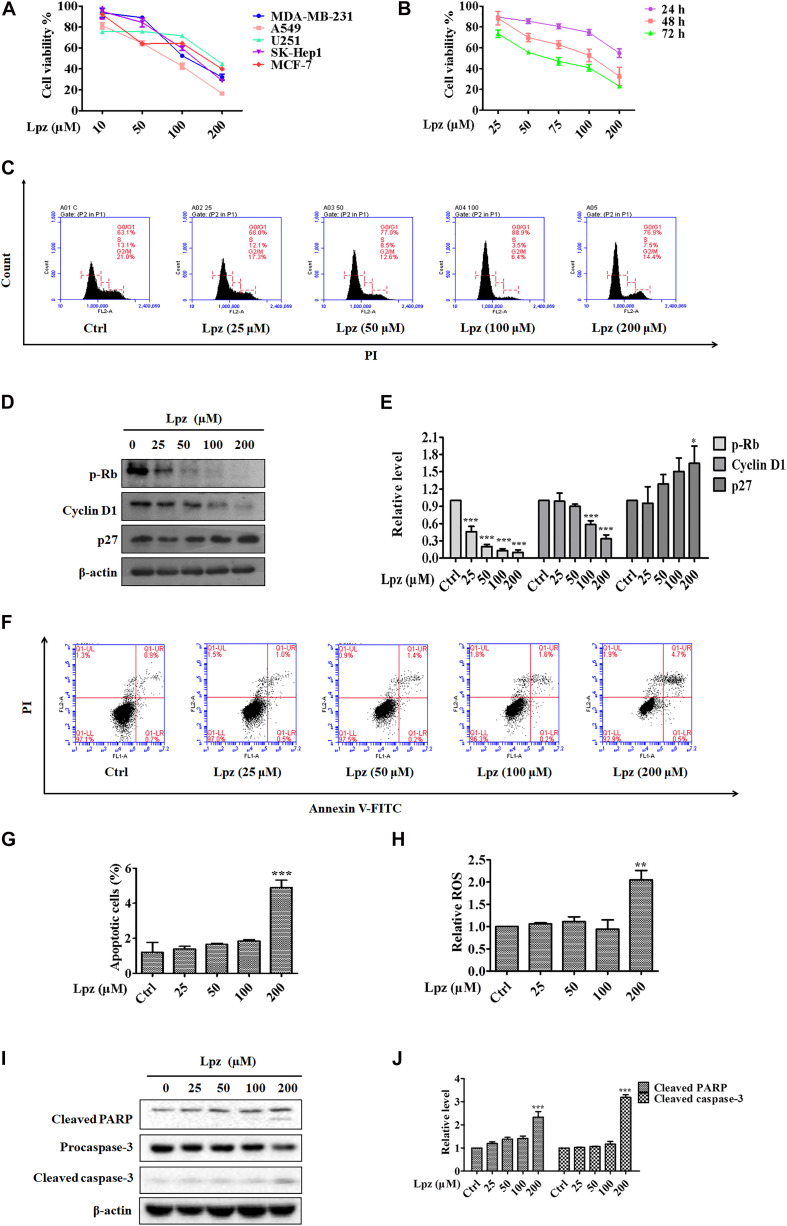
The antitumor effect of Lpz in A549 cells. **(A)** The antiproliferative effect of Lpz on a variety of cancer cells after 48 h. **(B)** Lpz inhibited A549 cell proliferation in a time- and concentration-dependent manner. **(C)** Lpz induced cell cycle arrest at G0/G1phase. **(D)** Effect of Lpz on G0/G1 cell cycle-related proteins. **(E)** Quantification of the results in panel **(D)**. **(F)** Lpz induced apoptosis in A549 cells. **(G)** The population analysis of the apoptotic cells. **(H)** Lpz enhanced intracellular ROS generation. **(I)** Lpz increased the levels of cleaved PARP and caspase-3. **(J)** Quantification of the results in panel **(I)**. Data shown are the mean ± SD of three independent experiments. *: *p* < 0.05, **: *p* < 0.01, and ***: *p* < 0.001.

First, we determined the dose responses to Lpz in different kinds of cancer cell lines, including MDA-MB-231 (human breast cancer), A549 (human NSCLC), U251 (human glioma), SK-Hep1 (human hepatocellular carcinoma), and MCF-7 (breast cancer), by MTT. As shown in Figure 1A, cancer cells were treated with Lpz for 48 h, and Lpz inhibited the proliferation of all tested cancer cells and showed the most potent antiproliferative activity in A549 cells. Therefore, we used A549 cancer cells to perform a subsequent antitumor mechanism study of Lpz. Next, A549 cells were treated with Lpz for 24, 48, and 72 h. The proliferation of A549 cells was significantly inhibited by Lpz in a dose- and time-dependent manner, with IC_50_ values of 110.4 and 69.5 μM at 48 and 72 h, respectively (Figure 1B). However, treatment with Lpz did not inhibit 50% of cell viability until 200 μM at 24 h.

Dysregulation of the cell cycle is a hallmark of cancer that leads to aberrant cellular proliferation. The cell cycle consists of sequential phases that go from quiescence (G0 phase) to proliferation (G1, S, G2, and M phases) and back to quiescence (Diaz-Moralli et al., 2013). To determine whether Lpz-induced growth inhibition was influenced by cell cycle arrest, A549 cells were incubated with or without Lpz for 48 h, and the cell cycle was examined through flow cytometry. Cell cycle arrest at G0/G1 phase was observed more frequently in the Lpz-treated A549 cells compared with the non-treated cells (Figure 1C). The relative percentages of G0/G1 phase cells progressively increased Lpz-treated A549 cells, respectively. p-Rb and a target of CDKs were involved in G1 and the restriction checkpoint. Cyclin D1 affects the function of p-Rb by binding with CDK4 and CDK6 (Wenzel and Singh, 2018). CDK activity is negatively regulated by p27. Therefore, the CDK4/6-cyclin D complex facilitates G1 progression. Western blot analysis also showed that Lpz treatment decreased p-Rb and cyclin D1 but increased p27 expression compared with non-treated cells (Figures 1D,E).

Next, the cell apoptosis-inducing effects were analyzed by flow cytometry with Annexin V/PI staining. Apoptosis is a form of programed cell death, and its molecular signaling pathway is well known. Figures 1F,G show increase in the proportions of apoptotic cells of 1.6, 1.5, 1.6, 1.8, and 5.2% after 0, 25, 50, 100, and 200 μM Lpz treatment, respectively. ROS are potent stimulators of apoptosis (Hayes et al., 2020), therefore, we investigated if Lpz increased ROS or not. A549 cells were treated with Lpz and the intracellular ROS levels were determined by flow cytometer. As indicated in Figure 1H, the level of ROS was dramatically enhanced after treatment with Lpz at 200 μM. The apoptosis pathway involves the activation of a series of caspases (Sankari et al., 2012). Apoptosomes (made up of cytochrome-C, apoptotic protease activating factor-1, and caspase-9) activate the downstream caspase-9/-3 signaling cascade and consequently result in apoptosis (Ou et al., 2017). Caspase-3 cleavage of PARP is a hallmark of apoptotic cell death (Cohen, 1997). Moreover, Western blot analysis showed that the expression levels of cleaved PARP and caspase-3 were distinctly higher in response to Lpz treatment than in the non-treated group (Figures 1I–J).

### Effect of Lpz on Migration in A549 Cells

**FIGURE 2:**
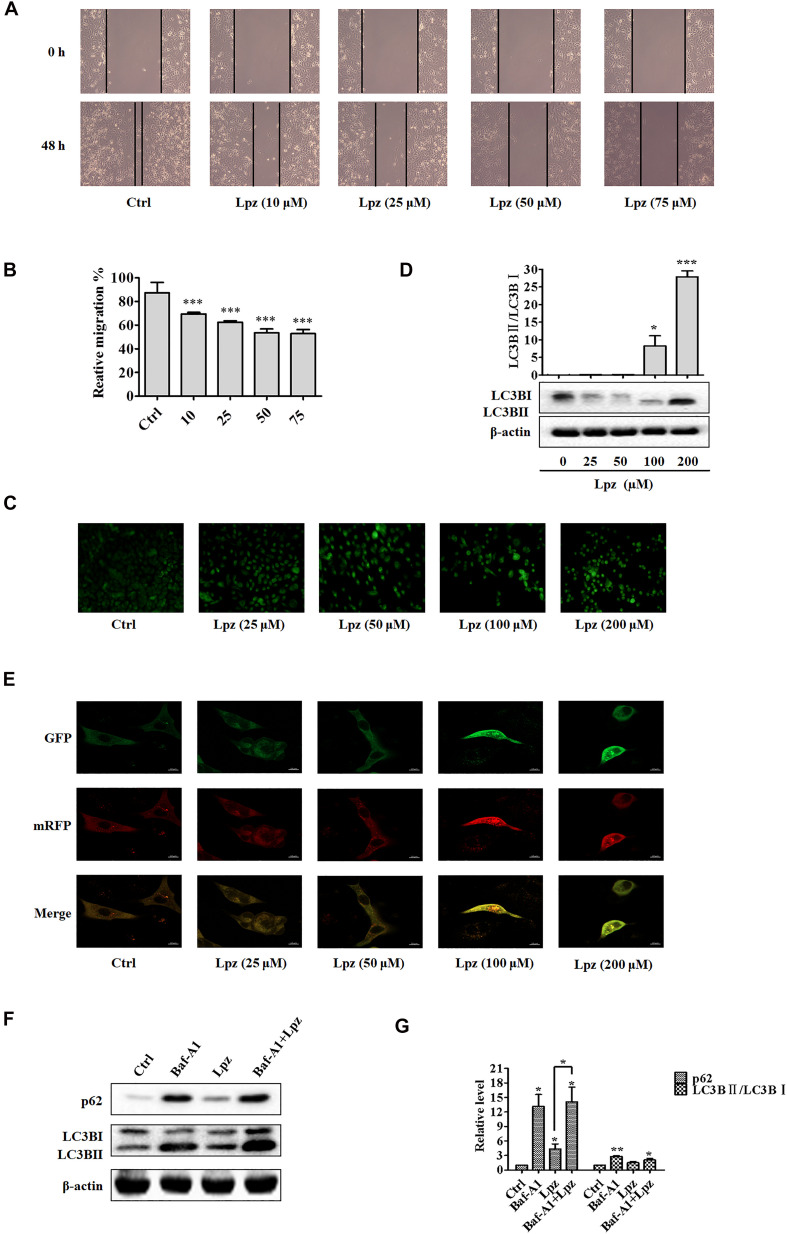
Lansoprazole inhibits migration and autophagy in A549 cells. **(A)** Representative images depicting the wound healing ability of A549 cells with or without Lpz treatment. **(B)** Quantification of the results in panel **(A)**. **(C)** Observation of autophagic vesicle formation by MDC staining. **(D)** Western blot analysis of the levels of LC3B in the absence or presence of Lpz. **(E)** Lpz suppresses autophagic flux in A549 cells. **(F)** Western blot analysis of the levels of p62 and LC3B in the presence of Lpz or Baf-A1 alone or in combination. **(G)** Quantification of the results in panel **(F)**. Data shown are the mean ± SD of three independent experiments. *: *p* < 0.05, **: *p* < 0.01, and ***: *p* < 0.001.

Next, a wound healing assay was performed to evaluate the role of Lpz in A549 cell migration. The cell mobility was reflected by wounded areas. After the scratches were formed, the cells were cultivated with different concentrations of Lpz (10, 25, 50, and 75 μM), pictures were taken at different time points at the same position (0 and 48 h), and the width of the scratch was calculated in A549 cells. To eliminate the possibility that cytotoxicity could influence migration, we chose concentrations lower than the IC_50_. It is important to highlight that cell migration was assessed at lower concentrations to avoid cytotoxic effects. Thus, we chose concentrations of 10, 25, 50, and 75 μM Lpz incubated with A549 cells. As illustrated in Figure 2A, the wound closure incidence was lower in the Lpz exposure group than in the control group (*p* < 0.001) (Figure 2B).

### Lpz Inhibits Autophagic Flux in A549 Cells

Autophagy describes the intracellular lysosomal degradation and recycling of proteins and organelles. To confirm whether Lpz affects autophagy in A549 cells, we determined the effect of Lpz on autophagy with various assays over 48 h. First, the number of autophagic vacuoles was detected in an MDC incorporation assay. Under MDC staining, the number of bright green fluorescent dots increased significantly after treatment with Lpz compared with non-treated cells (Figure 2C).

Next, we used Western blotting to investigate the conversion of LC3B I to LC3B II in control and Lpz-treated A549 cells. A549 cells were treated with different concentrations of Lpz for 48 h, and cell lysates were collected for immunoblot analysis with LC3B antibody. The increased level of LC3B II has been used to represent the extent of autophagy. As shown in Figure 2D, the conversion of LC3B I to LC3B II protein expression was increased by Lpz treatment in a concentration-dependent manner.

The dynamic process of autophagy consists of three parts: autophagosome formation, fusion of autophagosomes with lysosomes, and degradation (Zhang et al., 2013). To evaluate the dynamic influence of Lpz on the autophagic flux process, A549 cells were infected with mRFP (monomeric red fluorescent protein)-GFP (green fluorescent protein)-tagged LC3. The acidic pH inside the autolysosome quenches the fluorescent signal of GFP; in contrast, mRFP is not quenched in autolysosomes. Therefore, autophagosomes and autolysosomes are labeled with yellow (mRFP and GFP) and red (mRFP only) signals (“puncta”), respectively (Maulucci et al., 2015). Therefore, if most puncta exhibit both red and green signals, autophagy is impaired. Non-treated cells revealed few yellow dots. However, Lpz treatment led to an obvious increase in the number of yellow dots, and most of the green puncta were colocalized with red puncta (Figure 2E), indicating that Lpz inhibited autophagic flux in A549 cells in a concentration-dependent manner.

To further confirm that Lpz indeed attenuates autophagy, we further examined p62, a marker of autophagolysosomal levels, and the expression and conversion of LC3B I into LC3B II in control and Lpz-treated cells in the presence or absence of the specific V-ATPase inhibitor bafilomycin A1 (Baf-A1) by Western blot analysis. p62 is a selective autophagy substrate and is continuously degraded by autophagy (Johansen and Lamark, 2011). A549 cells were pre-treated with or without 0.1 μM Baf-A1 for 4 h and were then further treated with 100 μM Lpz for 4 h. Lpz treatment resulted in a significant increase in the p62 expression level compared with the control; Baf-A1 also increased the expression of p62. However, Lpz in combination with Baf-A1 treatment did not reverse the Baf-A1-induced conversion of LC3B I to LC3B II, and the level of p62 was non-significant (Figures 2F,G). These findings demonstrated that Lpz suppressed the fusion of autophagosomes with lysosomes.

### Effect of Lpz on the Phosphorylation of Stat3, PI3K/Akt, and the Raf/ERK Pathway in A549 Cells

**FIGURE 3:**
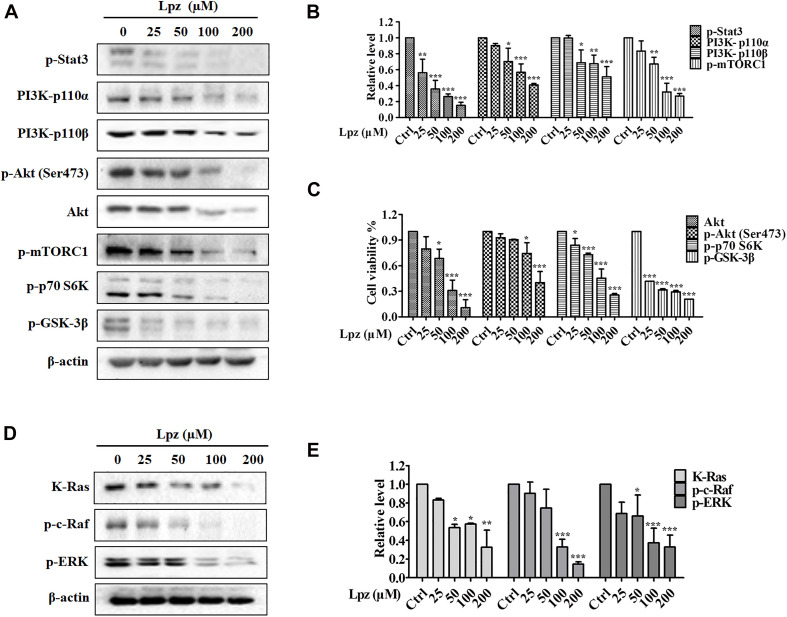
Lansoprazole inhibits phosphorylation of the Stat3, PI3K/Akt/mTOR, and Ras/Raf/ERK pathways. **(A)** Lpz downregulated the phosphorylation of Stat3, Akt, mTOR, p70 S6K, and GSK-3β and the expression of PI3K. **(B,C)** Quantification of the results in panel **(A)**. **(D)** Lpz suppressed the expression of K-Ras and the phosphorylation of c-Raf and ERK. **(E)** Quantification of the results in panel **(D)**. Data shown are the mean ± SD of three independent experiments. *: *p* < 0.05, **: *p* < 0.01, and ***: *p* < 0.001.

To verify the antitumor mechanism of Lpz, we detected the effect of Lpz on the phosphorylation of Stat3, PI3K/Akt, and the Raf/ERK pathway. Stat3 is an important class of transcription factors that have been implicated in a wide variety of essential biological processes, including cell cycle progression, survival and angiogenesis (Chai et al., 2016; Srivastava and DiGiovanni, 2016). Therefore, we examined the activation of Stat3 using Western blotting. The results showed that Stat3 was potently phosphorylated in non-treated A549 cells, while this phosphorylation of Stat3 was inhibited by Lpz treatment (Figures 3A,B).

The PI3K/Akt pathway is one of the major intracellular signaling pathways responsible for promoting cell survival. Therefore, we investigated the effect of Lpz on the PI3K/Akt pathway in A549 cells. The results showed that Lpz significantly reduced the levels of PI3K 110α and 110β, which are PI3K isoforms (Figures 3A,B). Furthermore, we examined the phosphorylation levels of PI3K downstream effectors, including Akt, mTORC1, p70 S6K, and GSK-3β. As shown in Figures 3A,C, the phosphorylation of Akt was markedly decreased by Lpz compared with the vehicle control. However, the total Akt level was also suppressed by Lpz treatment. In addition, the phosphorylation levels of mTORC1, p70 S6K, and GSK-3β were also downregulated in cells treated with Lpz.

The Ras/Raf/MEK/ERK pathway also plays a pivotal role in cell survival during various stages of cancer. We further validated the effect of Lpz on the Ras/Raf/ERK pathway in A549 cells. K-Ras level was depressed by Lpz treatment in a concentration-dependent manner. Concomitantly, the phosphorylation levels of Raf and ERK were also upregulated in non-treated A549 cells, and these levels were reduced by the addition of Lpz compared with non-treated cells (Figures 3D,E).

### Synergistic Effect of Lpz and Gef in A549 Cells *in vitro*

**FIGURE 4:**
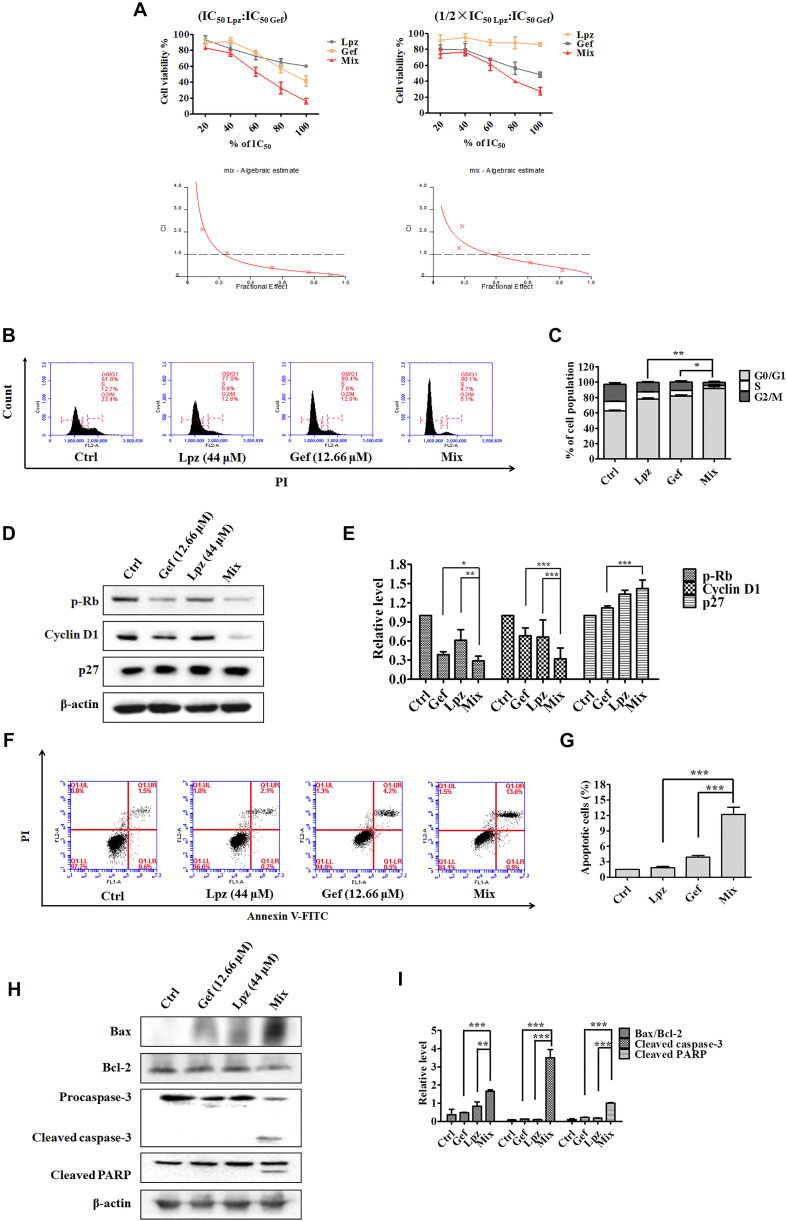
Antitumor effect of Lpz in combination with Gef on A549 cells. **(A)** A549 cells were treated with Lpz and Gef (20, 40, 60, 80, or 100% IC_50_ values of each drug), either alone or in combination for 48 h. In particular, in the combination groups, cells were pre-treated with Lpz for 2 h and were then treated with Gef for 48 h. Two fixed ratios of IC_50 *lansoprazole*_:IC_50 *gefitinib*_ and 1/2 × IC_50 *lansoprazole*_:IC_50 *gefitinib*_ were used. The combined effect was analyzed using CalcuSyn software, and the resulting CI-Fa plots are shown for A549 cells. **(B)** Lpz combined with Gef obviously arrested the A549 cell cycle in G0/G1 phase compared with either Lpz or Gef alone. **(C)** Quantification of the results in panel **(B)**. **(D)** Identification of the G0/G1-related proteins by Western blot. **(E)** Quantification of the results in panel **(D)**. **(F)** Lpz in combination with Gef facilitates A549 cell apoptosis. **(G)** Quantification of the results in panel **(F)**. **(H)** Identification of the apoptosis-related proteins by Western blot. **(I)** Quantification of the results in panel **(H)**. Data shown are the mean ± SD of three independent experiments. *: *p* < 0.05, **: *p* < 0.01, and ***: *p* < 0.001.

Lansoprazole has shown potent antitumor activity in A549 cells; thus, we asked whether the combination of Lpz and the current anticancer drug would produce synergistic antitumor activity in lung cancer. Currently, Gef is still the classical drug used in the clinic against NSCLC. Therefore, we next investigated whether Lpz could synergize with Gef in A549 cells. To analyze the synergistic effect, Chou and Talalay’s method (Chou, 2010) was used. As a first approach to test this hypothesis, we analyzed the antiproliferative effect of Gef in A549 cells. Cells were treated with Gef for 48 h, and Gef suppressed cell proliferation with an IC_50_ value of 15.83 μM (data not shown). Then, drug combinations were carried out by altering the ratio of Lpz to Gef (1 × IC_50 Lpz_: 1 × IC_50 Gef_; 1/2 × IC_50 Lpz_: 1 × IC_50 Gef_) and using a series of drug combinations (20, 40, 60, 80, and 100% of the IC_50_ value of each drug). In Lpz and Gef combinations, cells were pre-treated with Lpz for 2 h and were then treated with Gef for further 48 h. As shown in Figure 4A, the combination of Lpz and Gef when the ratio was 1 × IC_50 Lpz_: 1 × IC_50 Gef_ or 1/2 × IC_50 Lpz_: 1 × IC_50 Gef_ led to greater inhibitory effects of proliferation compared with drug alone. The combination index (CI), which describes the interaction between drugs, was analyzed, and the ED50, ED75, and ED90 values were calculated with CalcuSyn software. When CI < 1 at the indicated fraction affected (Fa) points, their combination exhibited synergism. To reduce the concentration of Lpz, we chose an 80% ratio of 1/2 × IC_50 Lpz_:1 × IC_50 Gef_. Therefore, the concentrations of Lpz and Gef used were 44 and 12.66 μM, respectively (Figure 4A).

To understand the action of these therapeutic strategies, we investigated the combined effect of Lpz and Gef on the cell cycle progression of A549 cells. As shown in Figures 4B,C, treatment with the combination of Lpz and Gef caused a significant increase in the proportion of cells in the G0/G1 phase compared with the Lpz or Gef alone group. Consistent with these results, the combination treatment significantly reduced the levels of phosphorylated Rb and cyclin D1, while the p27 level was increased compared with that of Lpz or Gef alone (Figures 4D,E).

Furthermore, Lpz or Gef alone did not potently increase the percentages of apoptotic cells, while treatment with a combination of Lpz and Gef significantly increased the percentages of apoptotic A549 cells (Figures 4F,G). There are two groups of Bcl-2 family proteins, pro- and antiapoptotic proteins, and cell health relies on the balance among these proapoptotic and antiapoptotic Bcl-2 proteins (Sankari et al., 2012; Goldar et al., 2015). As shown in Figures 4H,I, the combination of Lpz and Gef substantially enhanced the ratio of Bax (proapoptotic protein)/Bcl-2 and the levels of cleaved caspase-3 and cleaved PARP compared with treatment with Lpz or Gef alone.

**FIGURE 5:**
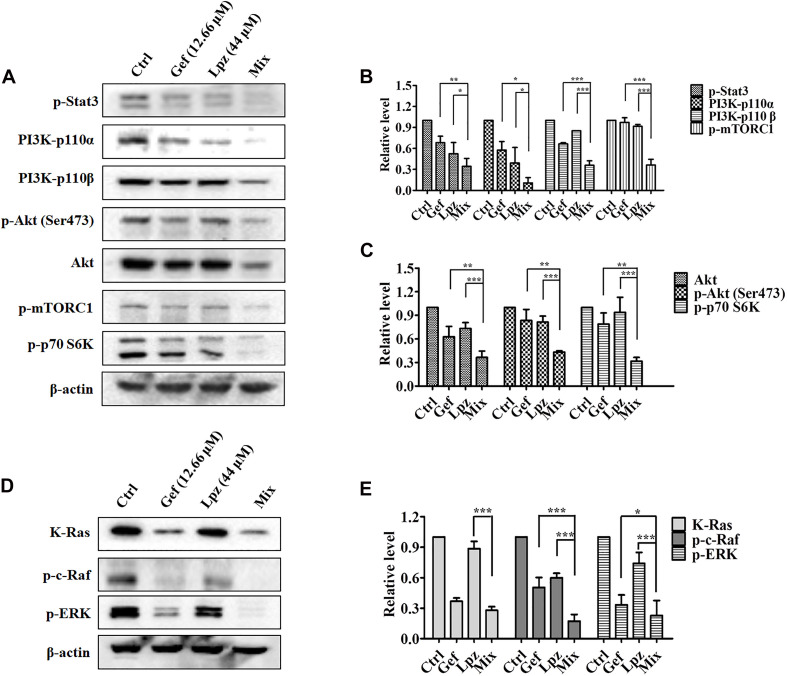
The combination of Lpz and Gef synergistically inhibits phosphorylation of the Stat3, PI3K/Akt/mTOR, and Ras/Raf/ERK pathways. **(A)** Lpz and Gef suppressed the phosphorylation of Stat3, Akt, mTOR, and p70 S6K and the expression of PI3K. **(B,C)** Quantification of the results in panel **(A)**. **(D)** Lpz and Gef suppressed the expression of K-Ras and phosphorylation of c-Raf and ERK. **(E)** Quantification of the results in panel **(D)**. Data shown are the mean ± SD of three independent experiments. *: *p* < 0.05, **: *p* < 0.01, and ***: *p* < 0.001.

We next examined the effect of the Lpz and Gef combination on the Stat3, PI3K, and Raf/ERK pathways. Western blot analysis revealed that Lpz in combination with Gef decreased Stat3 phosphorylation (Figures 5A,B). PI3K 110α and 110β were positively expressed in non-treated cells, whereas they were downregulated by Lpz in combination with Gef treatment. Furthermore, Lpz in combination with Gef suppressed Akt phosphorylation. However, the combination treatment had an evident influence on the total Akt. mTORC1 and p70 S6K, important molecules downstream of Akt, were found to be obviously decreased by Lpz in combination with Gef treatment (Figures 5A,C).

Furthermore, we also performed Western blotting to analyze the effect of Lpz in combination with Gef on the Raf/ERK pathway. As shown in Figures 5D,E, a markedly high expression of K-Ras in A549 cells was observed, but this expression significantly decreased to 27.5% after Lpz and Gef combination treatment. In addition, the combination treatment led to the downregulation of Raf and ERK phosphorylation compared with the Lpz or Gef alone group.

### Effect of Lpz Alone or in Combination With Gef on Tumor Growth *in vivo*

**FIGURE 6:**
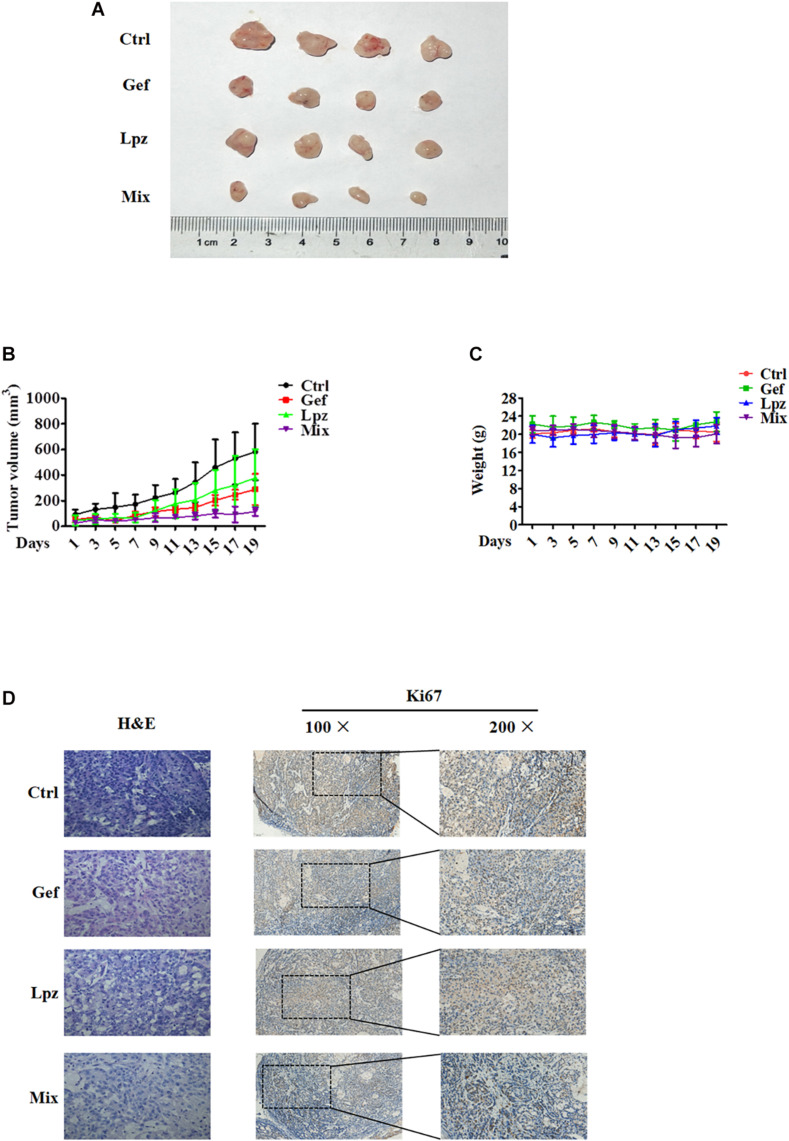
Lansoprazole in combination with Gef reduces the growth of A549 subcutaneous xenografts. Equal amounts of A549 cells were injected subcutaneously into nude mice. Once the tumor volume grew to 30–50 mm^3^, mice were randomized into four groups and treated with Lpz (25 mg/kg) or Gef (80 mg/kg) either alone or in combination (Lpz was administered orally 2 h before Gef) every other day for 19 days. **(A)** Representative images of the tumors removed from nude mice. **(B)** Quantification of tumor volume. **(C)** No significant differences in weight were found between the Lpz-, Gef-, or combination-treated groups. **(D)** Immunohistochemical staining of Ki67 in tumor tissues of different groups.

To further investigate the antitumor efficacy of Lpz in combination with Gef *in vivo*, we studied the effect of oral administration of Lpz and Gef in A549 cell-injected tumor xenografts. A549 cells were subcutaneously injected into six-week-old BALB/c nude mice. When tumors were approximately 30–50 mm^3^, mice were divided randomly into four groups of four animals, and drug intervention was initiated. Mice were orally administered 25 mg/kg Lpz, 80 mg/kg Gef, or the two in combination every other day. After 19 days of oral administration, mice were sacrificed, and representative tumor images are shown in Figure 6A. As shown in Figure 6B, treatment with Lpz inhibited the growth of lung tumors compared with untreated control xenografts, and combining Lpz and Gef decreased tumor growth compared with Lpz or Gef alone. Oral administration of Lpz or Gef did not change the mouse body weight (Figure 6C).

Hematoxylin and eosin (H&E) staining was performed to determine tissue morphology. Immunostaining of Ki67 was used to determine tumor cell proliferation. Lpz positively reduced tumor cell proliferation compared with the non-treated control group (Figure 6D). Furthermore, the antiproliferative effect was potentiated when mice were treated concomitantly with Lpz and Gef compared with the Lpz or Gef alone group.

## Discussion

Vacuolar-H^+^ ATPase mediates various functions in tumors. V-ATPase contributes to lower extracellular pH and activates extracellular metalloproteinases that promote tumor proliferation, motility and invasion, resulting in enhanced malignancy ability. Also, the abnormal pH gradient characterizing tumor cells is tuned by different ion/proton pumps such as vacuolar V-ATPase (Nishi and Forgac, 2002; De Milito et al., 2010). Pre-treatment of PPIs could inhibit V-ATPase and increase both extracellular pH and pH of lysosomal organelles (De Milito and Fais, 2005). The antitumor efficacy and mechanistic action of V-ATPase inhibitors have been reported in both human and murine models encompassing many tumor types; for example, omeprazole induces cell death of human B-cell tumors through severe alteration of pH gradient regulation, and the growth of human B-cell lymphomas in xenograft mice treated with omeprazole is significantly reduced compared with that in control animals (De Milito et al., 2007). Oral administration of V-ATPase inhibitors is efficacious in ameliorating osteolysis induced by metastatic breast cancer (Niikura, 2007), and inhibiting V-ATPase function through RNA interference can effectively retard cancer growth and prevent cancer metastasis by decreasing proton extrusion and downregulating gelatinase activity (Lu et al., 2005).

In this study, we investigated the antitumor activity of Lpz alone or in combination with Gef in A549 lung cancer cells. Lpz showed an excellent antitumor effect on A549 cells in our present work. The cell cycle is a complex sequence of events responsible for proper cell division into genetically identical daughter cells; therefore, the cell cycle is essential for cell growth and reproduction (Diaz-Moralli et al., 2013; Wenzel and Singh, 2018). Cells can enter the first gap phase G1 from the quiescent state G0. Our present results showed that Lpz treatment induced significant G0/G1 phase arrest in A549 cells. During G1 phase, D-type cyclins (D1, D2, and D3) promote CDK4 and CDK6 activation (Bonelli et al., 2019). D-type cyclins bind to CDK4/6, forming complexes that are stabilized by p27 (Goel et al., 2018). CDK4/6-cyclin D complexes enter the nucleus and phosphorylate Rb (Goel et al., 2018; Bonelli et al., 2019). We found that p-Rb and cyclin D1 were decreased after Lpz treatment, while p27 expression was elevated with Lpz treatment compared with the non-treated control group in the present study. Another interesting finding is the occurrence of apoptosis after Lpz treatment, and this efficacy was extreme when the concentration was 200 μM. In addition, Lpz potently increased level of intracellular ROS, particularly at 200 μM. Misbalancing of the fine-tuning between the levels of ROS and endogenous antioxidants could induce oxidative stress and, in worse conditions, apoptosis (Sinha et al., 2013). Apoptosis is recognized as the most important form of cell death and involves multiple factors. Induction of apoptosis is conducted by two main apoptotic pathways, including intrinsic and extrinsic pathways (Sankari et al., 2012). The intrinsic pathway is mitochondrial-mediated apoptosis, which is mediated by cytochrome C release and the activation of caspase-9 and caspase-3 (Goldar et al., 2015). PARP1 plays an important role in DNA repair (Morales et al., 2014); therefore, inhibition of PARP1 prevents DNA repair and leads to cell death. Western blot analysis also revealed that the cleavage of caspase-3 and PARP was upregulated after Lpz treatment. Connecting all these phenomena suggested that Lpz-mediated cell death involves cell cycle arrest and apoptosis.

After establishing the primary tumor and organized nutrition as well as protection against immune cell attacks, tumor cells have to acquire changes to migrate to distant sites and to establish metastasis (Popper, 2016). In our *in vitro* experiments, we found that treatment with Lpz decreased the migration of A549 cell monolayers. Therefore, these data indicate that Lpz plays an essential role in suppressing the migration of A549 cells.

Emerging evidence suggests that the dysregulation of autophagy has implications in a broad spectrum of human diseases, such as cancer (Zhang et al., 2013). Autophagy is a tightly orchestrated process that sequesters misfolded proteins, damaged or aged organelles, and mutated proteins in double-membrane vacuoles called autophagosomes that ultimately fuse with lysosomes, resulting in the degradation of sequestered content, known as autophagic cargo (Mowers et al., 2018; Onorati et al., 2018). MDCs can accumulate in mature autophagic vacuoles and are usually used to detect autophagic vacuoles. Lpz treatment resulted in an increase in MDC fluorescence in a concentration-dependent manner. In addition, the conversion of LC3B I to LC3B II was also elevated with Lpz treatment. LC3 II accumulation is a marker of autophagy. These results suggested that Lpz can increase the number of autophagic vacuoles in A549 cells. However, it was unclear whether this was due to enhanced autophagosome accumulation from increased autophagic flux or from decreased autophagic flux due to suppressed autophagosome clearance in the lysosome. It has been reported that pantoprazole, a PPI, appears to inhibit autophagy through a mechanism similar to Baf-A1 in PC3 cells (Tan et al., 2015). Baf-A1, as a potent and specific inhibitor of V-ATPase, prevents the maturation of autophagosomes into autolysosomes by suppressing fusion between autophagosomes and lysosomes (Yamamoto et al., 1998). To confirm the effect of Lpz on autophagy, we also monitored the autophagic flux morphologically traced with mRFP-GFP-LC3. If autophagic flux increases, both yellow and red puncta are increased; if autophagosome maturation into autolysosomes is blocked, only yellow puncta are increased (Maulucci et al., 2015). In the present study, we found that Lpz exposure led to potent blockade of autophagic flux in A549 cells. These results suggest that Lpz lead to the accumulation of autophagosomes by blocking the fusion of autophagosomes with lysosomes, possibly by impairing acidification of the luminal space of lysosomes by inhibiting V-ATPase, thereby suppressing autophagy. Degradation of p62 and LC3II could indicate autophagic flux. Our results revealed that p62 degradation was blocked by Lpz. Furthermore, we found that Baf-A1 in combination with Lpz did not change the Baf-A1-enhanced levels of p62 and LC3B II. The concurrent increases in LC3B II and p62 suggested a blockage of autophagic flux; therefore, it was concluded that Lpz could inhibit autophagy. These findings further suggested that Lpz has potent antitumor effects not only by inducing apoptosis and cell cycle arrest but also by diminishing cell migration and autophagy.

Persistently activated or tyrosine-phosphorylated Stat3 (p-Stat3) is found in 54% of NSCLC primary tumors, suggesting that Stat3 is a promising molecular target for lung cancer. Stat3 is considered to play a tumor-promoting role in NSCLC; for example, a Stat3 inhibitor significantly suppresses the growth of NSCLC tumors by promoting apoptosis and reducing angiogenesis and cell proliferation (Weerasinghe et al., 2007), and miR-98-5p promotes apoptosis and inhibits the migration of A549 cells by downregulating Stat3 expression (Liu et al., 2018). The Stat3 signaling pathway is a multicomponent cascade. It has been reported that Stat3, as a transcription factor, can promote the expression of cyclin D1. Herein, we present evidence showing that Stat3 phosphorylation was markedly reduced with Lpz treatment.

The PI3K/Akt/mTOR pathway is frequently activated in human cancers (Zhao et al., 2017). PI3K phosphorylates phosphatidylinositol 4,5-bisphosphate (PIP2) to phosphatidylinositol 3,4,5-triphosphate (PIP3), a second messenger that in turn activates Akt, a serine/threonine kinase (Thorpe et al., 2015). Class I PI3Ks are heterodimeric proteins that consist of a catalytic subunit and a regulatory subunit. There are four isoforms of the catalytic subunit: 110α, 110β, 110δ, and 110γ (Zhao and Vogt, 2008). Therefore, we first investigated the protein expression of upstream members of the PI3K pathway that affect downstream activity, including PI3K isoforms. In the present study, we found that Lpz significantly decreased the levels of PI3K 110α and 110β. Additionally, Lpz reduced both the phosphorylation and expression of Akt. Why Lpz attenuates the level of total Akt might be studied in further studies. Activated Akt promotes cell growth by phosphorylation of downstream mTORC1, which phosphorylates p70 S6K and eukaryotic initiation factor 4E binding protein 1 (4EBP1), leading to increased protein synthesis (Sarris et al., 2012; Zhao et al., 2017). We found that Lpz markedly suppressed the phosphorylation of mTORC1 and p70 S6K. Akt signals also regulate inactivation of GSK-3β (Zhao and Vogt, 2008). In addition, we found that Lpz reduced the phosphorylation of GSK-3β.

The Ras-regulated Raf-MEK-ERK signaling pathway is deregulated in a variety of cancers, and activation of the terminal kinases in the ERK1/2 cascade results in their accumulation in the nucleus, where they phosphorylate various transcription factors to stimulate or repress gene expression (Kidger et al., 2018). We found that the phosphorylation of c-Raf and ERK was reduced by treatment with Lpz. A549 is a K-RASmut cell line, and we found that the expression of K-Ras was effectively decreased with Lpz. These results demonstrated that Lpz inhibits the proliferation of A549 cells by inhibiting the phosphorylation of Stat3, PI3K/Akt and the Ras/Raf/ERK pathway.

Several studies have indicated that PPIs have promising activity to enhance sensitivity to anticancer drugs, such as Lpz (Lpz pre-treatment), which enhances the therapeutic effects of doxorubicin both by improving its distribution and increasing its activity in solid tumors (Yu et al., 2015); paclitaxel and the Lpz combination (Lpz pre-treatment), which was extremely efficient against metastatic melanoma cells (Azzarito et al., 2015); and the PPI drug esomeprazole, which sensitizes both human osteosarcoma cell lines and xenografts to cisplatin (Ferrari et al., 2013). Gef, approved for therapy of patients with advanced NSCLC, causes G1 arrest and induces apoptosis in A549 cells (Chang et al., 2004). Based on this theory, we investigated Lpz and Gef combination chemotherapy. Our study showed that combining Lpz and Gef represents a therapeutic strategy in A549 lung cancer both *in vitro* and *in vivo*. Treatment with Lpz is usually delivered as pre-treatment in most research papers, including the abovementioned reports. Therefore, we also pre-treated with Lpz in the present study. A549 cells were pre-treated for 2 h with Lpz and were then treated for an additional 48 h with Gef. We found that combined treatment with Lpz and Gef had a significantly greater efficacy against A549 cells than either drug alone. Furthermore, to obtain further insight into the mechanism of operation in solid lung cancers, we used Western blotting to determine the influence of Lpz and Gef in combination. We found that the combination of Lpz and Gef had a synergistic effect against the proliferation of A549 cells by triggering apoptosis and cell cycle arrest.

Consistent with the *in vitro* results, we also found therapeutic activity of Lpz in combination with Gef in human lung cancer xenograft models. Treatment with Lpz or Gef alone led to a reduction in the size of the tumors, and the effect was further enhanced when the two treatments were combined. In addition, immunohistochemical analysis of Ki67 showed that cancer cell proliferation was strikingly reduced upon combined administration of Lpz and Gef. Taken together, these results show that the anticancer efficacy of Lpz combined with Gef is greater than that of either drug used alone. In addition, all of the results further confirm that Lpz alone or in combination with Gef had positive inhibitory effects on the development and progression of NSCLC.

## Conclusion

In conclusion, the results of this study indicated that Lpz has an antitumor effect in A549 lung cancer by inducing apoptosis and cell cycle arrest, inhibiting migration, and suppressing autophagy by inhibiting the phosphorylation of Stat3 and reducing the activation of the PI3K/Akt and Raf/ERK pathways. Furthermore, Lpz in combination with Gef has shown more potent anticancer activity than either Lpz or Gef alone. Our results provide an experimental foundation for Lpz as a potential treatment for lung cancer.

## Data Availability Statement

The raw data supporting the conclusions of this article will be made available by the authors, without undue reservation.

## Ethics Statement

The animal study was reviewed and approved by Laboratory Animal Center of the Institute of Radiation Medicine, Chinese Academy of Medical Sciences.

## Author Contributions

MJ and DK designed the experiments and acquired funding for the study. XZ, NZ, YH, and XD performed the experiments. XP, WW, ZZ, RW, and YQ provided the technical assistances. XZ and MJ wrote the manuscript. DK edited the manuscript. All authors contributed to the article and approved the submitted version.

## Conflict of Interest

The authors declare that the research was conducted in the absence of any commercial or financial relationships that could be construed as a potential conflict of interest.
